# Acupuncture plus Rehabilitation for Unilateral Neglect after Stroke: A Systematic Review and Meta-Analysis

**DOI:** 10.1155/2020/5301568

**Published:** 2020-03-10

**Authors:** Yonghui Hou, Ying Liu, Minying Li, Baile Ning, Zehuai Wen, Wenbin Fu

**Affiliations:** ^1^The Second Affiliated Hospital of Guangzhou University of Chinese Medicine, Guangzhou, Guangdong, China; ^2^The First Hospital of Shijiazhuang City, Shijiazhuang, Hebei, China; ^3^National Center for Design Measurement and Evaluation in Clinical Research, Guangzhou University of Chinese Medicine, Guangzhou, Guangdong, China

## Abstract

**Objectives:**

To systematically assess the efficacy of acupuncture combined with rehabilitation on unilateral neglect after stroke.

**Methods:**

The Cochrane Library, PubMed, Excerpt Medical Database (EMBASE), China National Knowledge Infrastructure (CNKI), China Science and Technology Journal Database (VIP), Chinese Biomedical Literature Database (CBM), and Wan Fang databases were searched online for randomised controlled trials (RCTs) of acupuncture and its effects on unilateral neglect after stroke from their inception to September 2019. RCTs on acupuncture combined with rehabilitation in the experimental group for unilateral neglect compared with rehabilitation alone or rehabilitation plus sham acupuncture in the control group were included. Two authors separately screened the literature, extracted the data, and evaluated the quality of the included studies. Review Manager 5.3 software was used for the data analysis.

**Results:**

A total of 542 patients from nine RCTs were included. The meta-analysis showed that the experimental groups could significantly improve Fugl–Meyer Assessment (FMA) (MD = 11.54, 95% CI [9.54, 13.54], *P* < 0.00001) and the ability of daily living (SMD = 1.35, 95% CI [0.64, 2.07], *P* < 0.00001) and the ability of daily living (SMD = 1.35, 95% CI [0.64, 2.07], *P* < 0.00001) when compared with the control groups. However, there was no significant difference in the drop of Catherine Bergego Scale (CBS) and Behavioural Inattention Test-conventional (BIT-C) between the two groups.

**Conclusions:**

Acupuncture combined with rehabilitation was more effective in improving the motor function and the ability of daily living. Because of the limitations regarding the quantity and quality of the studies in this meta-analysis, high-quality and well-designed RCTs are necessary to validate the above conclusions.

## 1. Introduction

Worldwide, stroke is responsible for increasingly high rates of mortality and disability [1]. Unilateral neglect is a frequent poststroke disorder that is characterised by the inability to detect, respond, or orient towards a contralesional stimuli [2, 3]. The presence of unilateral neglect significantly delays the recovery of hemiparesis, and patients often experience more problems with the activities of daily living (ADL) [4]. Unilateral neglect is also associated with falls, longer stays in rehabilitation, and need for more assistance at discharge [5].

Currently, in clinical studies, there are two types of outcomes that are used to evaluate the efficacy of certain measures in the treatment of unilateral neglect. First, the outcome can directly evaluate the degree of unilateral neglect, such as the Catherine Bergego Scale (CBS) and Behavioural Inattention Test-conventional (BIT-C) [6]. Second, these outcomes of motor function and activities of daily living are heavily influenced by unilateral neglect and can indirectly reflect the degree of this neglect, such as when measured by the Fugl–Meyer assessment (FMA), Modified Barthel Index (MBI), Barthel Index (BI), and Functional Independence Measure (FIM) [7, 8].

Many treatments have already been developed for the rehabilitation of unilateral neglect [5, 9]. However, a Cochrane systematic review and meta-analysis from 2013 concluded that there is not yet enough substantial evidence to support one cognitive rehabilitation intervention over another for the treatment of unilateral neglect to reduce the degree of neglect (CBS or BIT-C) or to increase the motor function (FMA) and activities of daily living (BI, MBI, or FIM) [10]. Similarly, another systematic review and meta-analysis from 2016 revealed novel interventions, such as sensory manipulations, prism adaptation, noninvasive brain stimulation, virtual reality, and pharmacological agents, need to be validated in a larger number of patients before any conclusions can be drawn about their effectiveness [8]. Hence, currently, it is not possible to formally recommend one rehabilitation method over another [8].

In reviewing the literature, it is evident that acupuncture is widely used for unilateral neglect treatment in China. However, no published meta-analysis has concluded whether acupuncture for unilateral neglect is effective and safe. Thus, the aim of the current systematic review and meta-analysis is to evaluate the efficacy and safety of acupuncture for the treatment of unilateral neglect.

## 2. Methods

### 2.1. Protocol and Registration

The protocol was registered at the International Prospective Register of Systematic Reviews (PROSPERO, the identification number: CRD42018087894).

### 2.2. Data Sources and Searches

The following electronic databases were searched to identify the relevant studies for inclusion in the review from their inception to September 2019: the Cochrane Library, PubMed, Excerpta Medica Database (EMBASE), China National Knowledge Infrastructure (CNKI), China Science and Technology Journal Database (VIP), Chinese Biomedical Literature Database (CBM), and Wan Fang databases. The search terms used were “Cerebrovascular Disorders” OR “Brain Ischemia” OR “Cerebral Hemorrhage” OR “stroke” and “unilateral neglect” or “hemispatial neglect” or “visuospatial neglect” or “unilateral spatial neglect” and “acupuncture” or “electroacupuncture” or “electro-acupuncture”. This search strategy for each database was adjusted, PubMed, for example (Appendix 1).

### 2.3. Inclusion and Exclusion Criteria

The relevant articles were selected according to following criteria: (1) the included studies were randomised controlled trials (RCTs) studying acupuncture for treating unilateral neglect after stroke; (2) the included participants had to be clinically diagnosed with unilateral neglect, and stroke was confirmed by computerized tomographic scan or magnetic resonance imaging; (3) studies in which the experimental group were given acupuncture (including acupuncture, electroacupuncture, or electro-acupuncture) combined with rehabilitation, while the control group underwent rehabilitation alone or rehabilitation plus sham acupuncture; (4) the primary outcome measures included CBS, BIT-C, FMA, BI, or MBI; (5) the secondary outcome was an analysis of adverse events (AEs) as a way to assess safety; and (6) full text should be accessible.

Articles with the following criteria were excluded: (1) non-RCTs; (2) duplicate studies with the same results; (3) the control group given acupuncture treatment; and (4) noninvasive brain stimulation used in the trials, such as repetitive transcranial magnetic stimulation (rTMS) or transcranial direct current stimulation (tDCS).

### 2.4. Screening Study and Data Extraction

Two independent researchers (Hou and Liu) screened all the studies by their titles and abstracts and removed the studies that did not meet the predefined eligibility criteria. If there were any disagreements during this process, the third researcher (Wen) was consulted. The following data were extracted from the included studies by two independent researchers (Hou and Liu): author, publication sample size, treatment period, interventions, outcomes, and adverse events. Two review authors (Hou and Liu) will independently extract data from the included trials, using a predesigned data extraction form. We will obtain additional information required by contacting the trial authors at least within one month if necessary, and we will include the information obtained in the review.

### 2.5. Risk of Bias Assessment

The Cochrane risk of bias assessment tool [11] was used to grade the risk of bias of the selected RCTs; this was carried out by two independent researchers (Hou and Liu). This tool has seven domains: random sequence generation, allocation concealment, blinding of participants and personnel, blinding of outcome assessments, incomplete outcome data, selective reporting, and other biases. Three categories (low risk of bias, high risk of bias, or unclear) were used to rank the risk of bias for each domain. Disagreements between the two researchers during the process were resolved through a discussion with the third researcher (Wen).

### 2.6. Data Analyses

Statistical analysis was performed by using RevMan 5.3 software (the Nordic Cochrane Centre, Cochrane Collaboration 2014). Because all the outcomes were continuous data, a mean difference (MD) with a 95% confidence interval (CI) was calculated for the CBS, BIT-C, FMA, BI, and MBI. A chi-squared test and Higgins *I*^2^ test were used to evaluate heterogeneity among the studies. When *I*^2^ was less than 50% or *P* was larger than 0.10, a fixed effects model was used; otherwise, a random effect model was used after excluding clinical heterogeneity. A sensitivity analysis by stratifying studies was performed to explore the impact of risk of bias on the pooled estimate and to investigate potential methodological heterogeneity. We will perform the analysis of reporting bias by funnel plot if the included trials are more than 10.

## 3. Results

### 3.1. Study Selection

**Figure 1:**
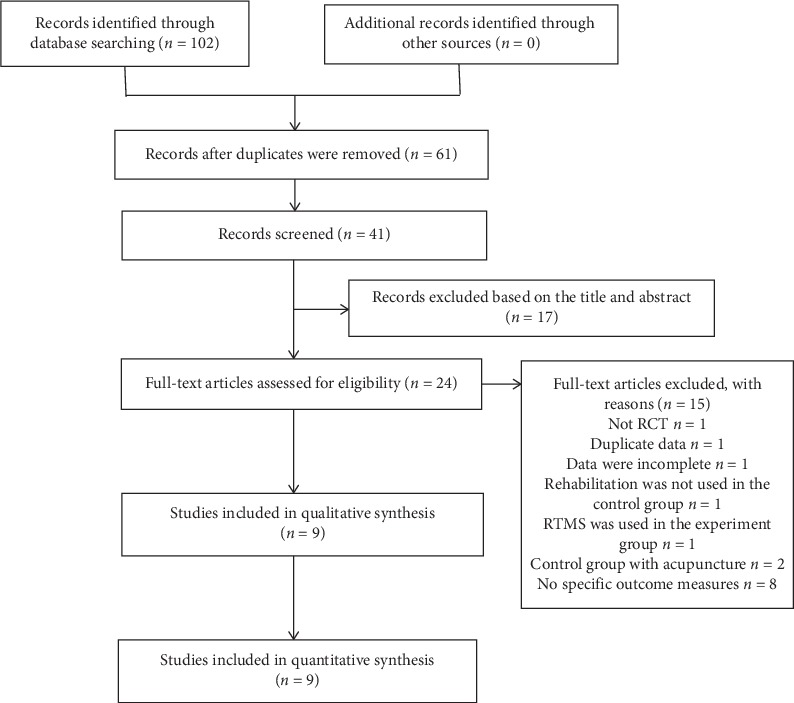
Flow chart for study selection.

A total of 102 studies were identified, and 61 duplicated articles were removed. Seventeen irrelevant studies were excluded after reviewing the titles and abstracts. The full texts of 24 studies were carefully read, of which 15 studies were excluded, including one study that was not a RCT, one duplicate study, one study with incomplete data, one study without rehabilitation treatment in the control group, one study with rTMS in the control group, two studies with acupuncture in the control groups, and eight studies without specific outcome measures. Finally, nine RCTs [12–20] were included in the quantitative analysis. In Figure 1, a flow chart shows the search process for study identification and selection.

### 3.2. Study Characteristics

The characteristics of all the included studies are presented in Table 1. All included studies are published from 2011 to 2017, and all of the studies originated from China. The publication language is Chinese. The sample size ranged from 14 to 50 participants. The treatment lengths of the studies varied from four to eight weeks. In the control groups, rehabilitation alone was used in all of the studies. In the experiment groups, rehabilitation was the base intervention, the other interventions included body acupuncture plus scalp acupuncture in five studies [12, 13, 15, 18, 20], body acupuncture plus electroacupuncture in two studies [14, 16], electroacupuncture plus rehabilitation in one study [19], and body acupuncture plus rehabilitation in one study [17].

### 3.3. Risk of Bias Assessment of the Included Studies

**Figure 2:**
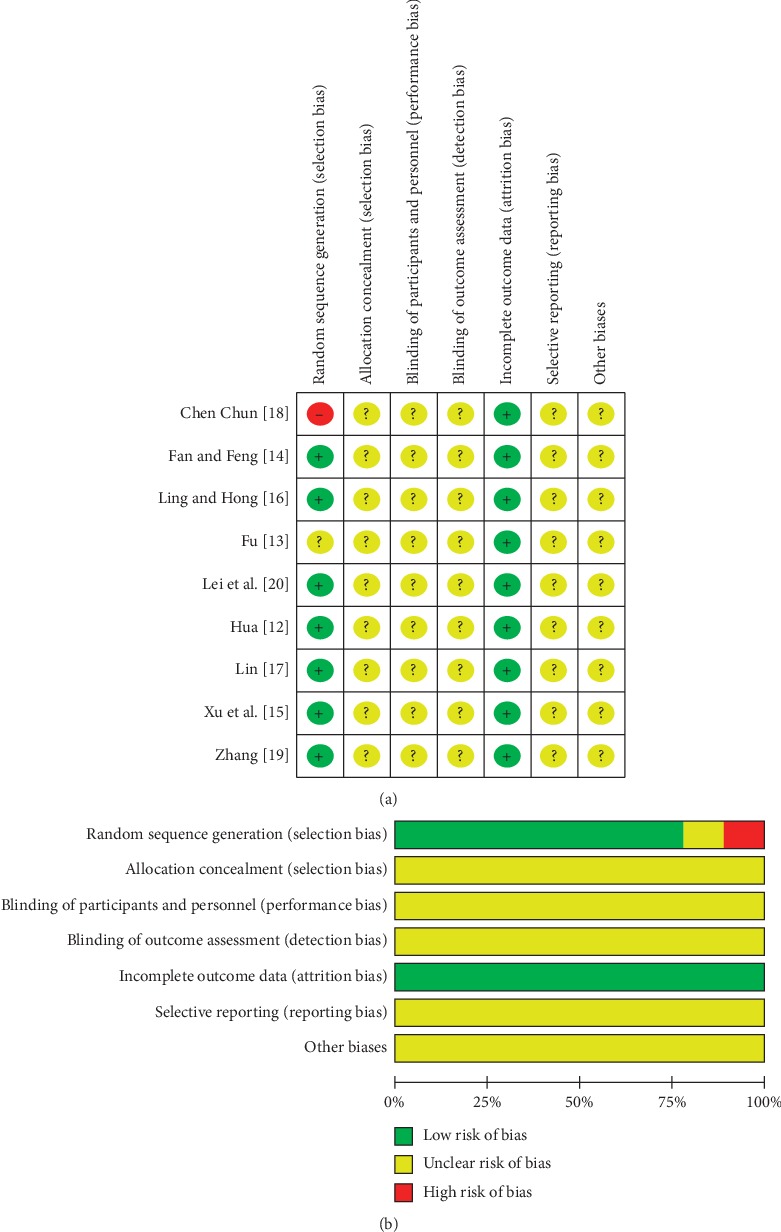
(a) Risk of bias summary. (b) Risk of bias graph.

The risk of bias of the selected studies is shown in Figure 2. Seven studies [12, 14–17, 19, 20] had adopted a random number table to allocate the treatment. One study [18] had applied the treatment order to divide its participants. One study [13] did not report the method of sequence generation. No study mentioned the use of allocation concealment. None of the included studies reported blinding the participants and practitioners. Regarding incomplete data, the studies were judged to have a low risk of bias because eight studies had no missing outcome data; another study [13] had missing outcome data, but the dropout rate was less than 20%. Selective reporting was unclear in all of the studies.

### 3.4. Outcomes Assessment

#### 3.4.1. FMA

**Figure 3:**
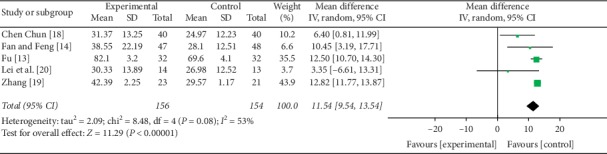
Forest plot of FMA.

Five studies [13, 16, 18–20] presented acupuncture's effects on motor function, measured by FMA. A random effect model was used, with *P* < 0.0001 and *I*^2^ = 53%. The meta-analysis indicated that acupuncture plus rehabilitation improved the FMA performance more than rehabilitation alone (MD = 11.54, 95% CI [9.54, 13.54], *P* < 0.00001) (Figure 3).

#### 3.4.2. Activity of Daily Live

**Figure 4:**
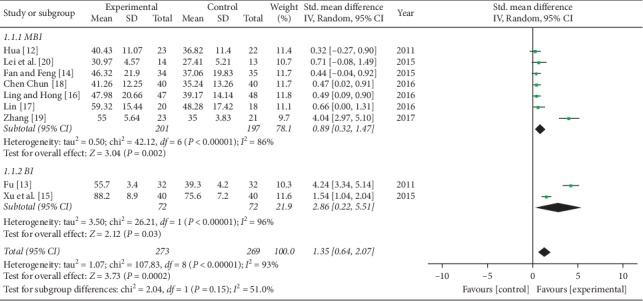
Forest plot of activity of daily living.

Nine studies presented acupuncture's effects on the activity of daily life performance, as measured by the BI or MBI, including seven with MBI and two with BI. MBI and BI measured the same functional activity of daily living, should be pooled by standardized mean difference (SMD). The random effect model was used with *I*^2^ = 93% and *P* < 0.00001. A meta-analysis indicated that acupuncture plus rehabilitation improved the activity of daily living performance more than rehabilitation alone (SMD = 1.35, 95% CI [0.64, 2.07], *P* < 0.00001) (Figure 4).

#### 3.4.3. CBS

**Figure 5:**
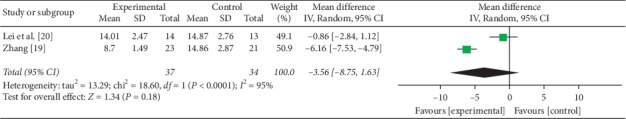
Forest plot of CBS.

Two articles [19, 20] presented acupuncture's effects on CBS. The random effect model was used, with *P* < 0.0001 and *I*^2^ = 95%. The meta-analysis indicated that there was no statistically significant difference between the two groups (MD = −3.56, 95% CI [−8.75, 1.63], *P* < 0.0001) (Figure 5).

#### 3.4.4. BIT-C

One article [17] assessed the effect of acupuncture plus rehabilitation compared with rehabilitation alone. Theresult indicated that there was no statistically significant difference between the two groups.

#### 3.4.5. Adverse Events

Eight articles did not report any details about adverse events. Only one study [15] reported that no adverse events occurred.

### 3.5. Sensitivity Analysis and Publication Bias

To test the sensitivity, the included studies were excluded one by one, and the results were proved to be stable. Because the number of included studies was insufficient, we did not perform the analysis of reporting bias by funnel plot.

## 4. Discussion

To the best of our knowledge, this is the first systematic review and meta-analysis that evaluates the efficacy of acupuncture for the treatment of unilateral neglect; nine studies that included a total of 542 patients were selected in our research. The findings indicate that acupuncture plus rehabilitation was more effective in the improvement of motor function and activity of daily living when compared with rehabilitation alone, but there was no statistical difference regarding the CBS and BIT-C. Eight studies did not report any details about the adverse events of acupuncture, and one study [15] documented that no adverse events occurred. This showed that the adverse events report was insufficient for safety assessment.

The World Health Organization's (WHO's) International Classification of Functioning, Disability, and Health (ICF) model provides an important framework of terminology for understanding and categorizing health conditions and patient problems by clearly defining health condition, impairment, activity limitation, and participation restriction [21]. According to the ICF model, unilateral neglect and motor function belong to the category of body functions, while the activity of daily life belongs to the category of activities. The improvement of motor function is the basis for improving daily life ability. Unilateral neglect leads to poor motor function recovery and important limitations to perform activities of daily living [22]. In the current paper, an interesting conclusion is drawn: when compared with rehabilitation alone, acupuncture plus rehabilitation improves the motor function and daily living without reducing the degree of unilateral neglect.

The mechanism for the effects of acupuncture in the improvement of motor function and activity of daily life may be explained by two reasons. First, acupuncture can directly enhance the motor function and daily life of stroke patients. Acupuncture has been shown to lead to a higher improvement in motor function than conventional treatment [23]. Acupuncture when combined with rehabilitation may have a positive effect on motor function, activity of daily life, neurological deficits, and spasticity [24]. Indeed, the meta-analysis [25] concluded that no matter what kind of acupuncture therapy was combined with rehabilitation, it was better than the rehabilitation group when it came to significantly improving the activity of daily life of patients after ischaemic stroke. Second, acupuncture may indirectly improve the motor function and daily living ability of stroke patients by improving the symptoms of unilateral neglect and making them aware of their limbs. Acupuncture points may be the more intensive parts of the sensory organs that produce needle sensation [26]. Acupuncture sensation is continuously transmitted to the central nervous system through the peripheral receptors of acupuncture point of contact, which facilitates new cognitive neural pathways for effective information, processing, and analysis, which may be one of the mechanisms for improving the perception of an injured hemisphere and alleviating the symptoms of unilateral neglect in patients [27].

However, there were several limitations to the current review. (1) All the studies did not mention allocation concealment, blinding of the participants and personnel, or blinding of the outcome assessments. Additionally, all of the included studies had small sample sizes, and none documented the calculation of their sample size. (2) Acupuncture therapy is not differentiated in detail, and rehabilitation therapy as a basic treatment may be different from study to study, which may result in unreasonable data consolidation. (3) Among the nine included studies, only one reported the dropout number and reasons for missing data. Because none of the other studies reported missing data, the effects of acupuncture for unilateral neglect might be exaggerated.

## 5. Conclusion

In conclusion, acupuncture and rehabilitation was more effective in the improvement of motor function and activity of daily living when compared with rehabilitation alone. However, considering the poor methodological quality of the articles, caution needs to be taken in utilising the conclusions of the present review. Therefore, further trials with a high methodological quality and larger sample sizes are necessary to verify the conclusions of the current review. It is also recommended to use CBS or BIT-C to assess the severity of unilateral neglect.

## Figures and Tables

**Table 1:** Characteristics of the included studies.

Study	Study location	Sample size (E/C)	Gender (male/female)		Mean age (years)		Duration after stroke (days)		Treatment period	Interventions		Outcomes	Adverse events
			E	C	E	C	E	C		E	C		
Hua [12]	China	23/22	18/5	16/6	62.35 ± 10.14	64.00 ± 8.88	70.96 ± 37.06	76.41 ± 36.71	8 weeks	R + A	R	MBI	NR
Fu [13]	China	32/32	—	—	—	—	—	—	8 weeks	R + A	R	BI, FMA	NR
Fan and Feng [14]	China	34/35	19/15	18/17	66.65 ± 11.19	57.28 ± 13.35	19.70 ± 3.38	32.88 ± 4.04	4 weeks	R + A	R	MBI	NR
Xu et al. [15]	China	40/40	29/11	32/8	63.10 ± 7.50	62.50 ± 7.60	—	—	8 months	R + A	R	BI	None
Lei et al. [20]	China	14/13	8/6	8/5	63.15 ± 11.24	63.00 ± 9.97	72.21 ± 36.86	75.15 ± 39.20	8 weeks	R + A	R	MBI, CBS, FMA	NR
Ling and Hong[16]	China	50/50	27/23	29/21	67.30 ± 9.80	60.90 ± 12.30	27.00 ± 18.40	30.40 ± 21.12	4 weeks	R + A	R	MBI, FMA	NR
Lin [17]	China	20/18	13/7	13/5	59.00 ± 8.00	60.00 ± 8.00	115.70 ± 31.30	109.40 ± 33.40	6 weeks	R + A	R	MBI, BIT-C	NR
Chen Chun [18]	China	40/40	—	—	—	—	—	—	8 weeks	R + A	R	MBI, FMA	NR
Zhang [19]	China	23/21	13/10	10/11	58.70 ± 5.20	59.40 ± 6.10	45.30 ± 5.40	43.70 ± 5.60	4 weeks	R + A	R	MBI, CBS, FMA	NR

E: experimental group; C: control group; A: acupuncture; R: rehabilitation; BIT-C: behavioural inattention test-conventional; CBS: Catherine Bergego scale; FMA: Fugl–Meyer assessment; BI: Barthel index; MBI: modified Barthel index; NR = not reported.

## Data Availability

The data supporting this systematic review are from the previous studies and datasets, which have been cited. The processed data are available from the corresponding author upon request.

## Appendix

### The Search Strategy of PubMed

1. “Cerebrovascular Disorders “[Tiab] OR” Brain Ischemia “[Tiab] OR” Cerebral Hemorrhage “[Tiab] OR” stroke” [Tiab] 246458
2. “Cerebrovascular disorders “[MeSH Terms] OR “brain ischemia” [MeSH Terms] OR “cerebral hemorrhage” [MeSH Terms] OR “stroke” [MeSH Terms] 359204
3. 1or 2 471999
4. “Acupuncture “[Tiab] OR” electroacupuncture “[Tiab] OR” electro-acupuncture” [Tiab] 23984
5. “Acupuncture” [MeSH Terms] OR “acupuncture therapy” [MeSH Terms] OR “electroacupuncture” [MeSH Terms] 24571
6. 4or 5 30064
7. 3 and 6 1637
8. “Unilateral neglect “[Tiab] OR” hemispatial neglect “[Tiab] OR” visuospatial neglect” [Tiab] OR
9. “unilateral spatial neglect” [Tiab] 1708
10. “Unilateral neglect “[MeSH Terms] OR” hemispatial neglect “[MeSH Terms] OR” visuospatial neglect “[MeSH Terms] OR” unilateral spatial neglect”[MeSH Terms] 27873
11. 8or 9 28689
12. 10 and 7 2
